# Risk Factors of Optic Neuropathy in Ethambutol Users: Interaction with Isoniazid and Other Associated Conditions of Toxic Optic Neuropathy

**DOI:** 10.3390/toxics12080549

**Published:** 2024-07-30

**Authors:** Jiyeong Kim, Seong Joon Ahn

**Affiliations:** 1 Department of Pre-Medicine, College of Medicine, and Biostatistics Lab, Medical Research Collaborating Center (MRCC), Hanyang University, Seoul 04763, Republic of Korea; kimzi@hanyang.ac.kr; 2 Department of Ophthalmology, Hanyang University Hospital, Hanyang University College of Medicine, Seoul 04763, Republic of Korea

**Keywords:** ethambutol, optic neuropathy, risk factors

## Abstract

(1) Background: To investigate the risk factors associated with optic neuropathy (ON) and validate the hypothesis that concomitant isoniazid use and other causes of toxic ON affect the development of ON in ethambutol users. (2) Methods: This cohort study identified ethambutol users who initiated ethambutol therapy between January 2015 and December 2021 and had no ON prior to ethambutol therapy. ON incidence up to 31 December 2022 was evaluated. The users were grouped on the basis of the presence of ON. Demographic and clinical characteristics were investigated for risk factor analyses of ON. Odds ratios (ORs) were calculated using multivariate logistic regression analyses. (3) Results: Among 204,598 ethambutol users, 5277 (2.6%) patients developed ON over the study period. Patients with ON included a higher percentage of women and had a higher mean age than patients without ON. In the multivariate analyses, the risk factors for ON and visual impairment included sex, age, cumulative dose, extrapulmonary indications for ethambutol use, and systemic conditions such as diabetes, hypertension, hyperlipidemia, diabetes, kidney disease, and liver disease. Malnutrition or nutritional disorders significantly increased the risk of ON (OR = 1.27, 95% confidence interval [CI] = 1.19–1.34), whereas concomitant isoniazid use decreased the risk (OR = 0.78, 95% CI = 0.72–0.86). (4) Conclusion: An increased risk of ON in patients with systemic diseases and nutritional deficiency was identified, whereas concomitant isoniazid use was associated with a decreased risk of ON. Patients with these risk factors should be carefully monitored to minimize the vision-threatening ON.

## 1. Introduction

Ethambutol, a key anti-tuberculosis medication, can induce optic neuropathy (ON), potentially leading to significant vision loss [1,2]. This adverse effect, known as ethambutol-induced ON (EON), often manifests as painless, progressive vision loss and can result in irreversible blindness if not promptly detected and managed [1,2]. Studies report that ON incidence ranges from 0.5% to 6% among ethambutol users, with older age, renal impairment, and daily dose >15 mg/kg implicated as potential risk factors [1,2,3,4,5]. The concomitant use of other anti-tuberculosis drugs, such as isoniazid, has been suggested as a contributing factor [6].

Isoniazid, another cornerstone of tuberculosis therapy, is known for its neurotoxic potential, which primarily manifests as peripheral neuropathy [7]. This side effect is attributed to the drug’s interference with pyridoxine (vitamin B6) metabolism, leading to a functional deficiency in the nerves [8]. Pyridoxine is essential for neurotransmitter synthesis, and its depletion can result in various neurological diseases such as peripheral neuropathy [8,9]. Although isoniazid is more commonly associated with peripheral neuropathy, there is growing concern that its neurotoxic effects might also affect the optic nerve, thus potentially compounding the risk of EON when isoniazid is used in combination with ethambutol [10].

However, ON can stem from various sources, including exposure to toxic substances and nutritional deficiencies [11]. For instance, alcohol consumption is linked to toxic ON and may exacerbate the risk of EON because of its metabolic effects [12]. Heavy smoking and nutritional deficiencies, such as vitamin deficiencies, are also associated with ON [11,13]. The prolonged use of ethambutol has been linked to deficiencies in vitamins E and B1, potentially exacerbating optic neuropathy by interacting with the nutritional cause of optic nerve damage [11]. Additionally, the combination of ethambutol with isoniazid and other nutritional or systemic factors that lead to toxic ON may further complicate ON development [1,6], making considering these interactions crucial to clinical management. Several studies suggest that the concurrent use of ethambutol and isoniazid and the presence of other risk factors, such as metabolic or nutritional deficiencies, could increase the susceptibility to ON [6,10,14], although the evidence is inconclusive, highlighting the need for careful monitoring and early intervention.

Given these associations, we hypothesized that concomitant isoniazid use and metabolic/nutritional deficiencies could serve as risk factors for EON, although this association has not been validated. Our study aims to validate this hypothesis by exploring systemic diseases/comorbidity data in a nationwide cohort of ethambutol users in South Korea by using a national health claims database. We specifically focused on understanding the interplay between ethambutol, isoniazid, and other potential conditions associated with ON.

## 2. Materials and Methods

### 2.1. Study Design and Population

This nationwide cohort study utilized the Health Insurance Review and Assessment Service (HIRA) database, which is a health claims database in South Korea covering approximately 50 million individuals (97% of the overall population). The database comprises comprehensive data on medication records, demographic details, and medical diagnoses utilizing the Korean Standard Classification of Diseases (either the seventh or eighth revision), which is slightly modified from the International Statistical Classification of Diseases and Related Health Problems, Tenth Revision (ICD-10) [15].

**Figure 1:**
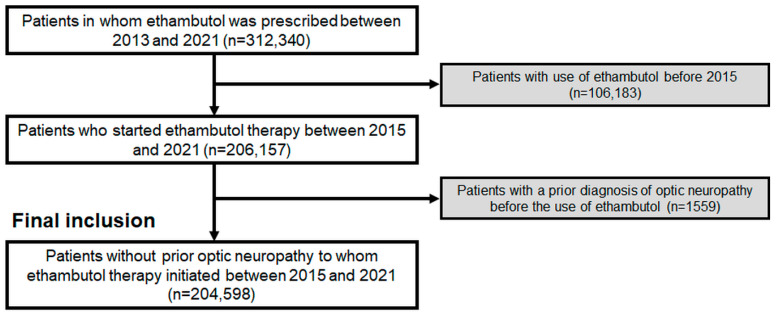
A flowchart of the study population and inclusion/exclusion criteria in this study.

Ethambutol users who initiated therapy between 1 January 2013 and 31 December 2021 were identified for inclusion in the study cohort. To focus on users who began the drug between 1 January 2015 and 31 December 2021, we excluded those receiving an ethambutol prescription between 1 January 2013 and 31 December 2014. This exclusion criterion was based on the assumption that the absence of ethambutol prescriptions for two years prior to 2015 indicates no history of ethambutol use before 2015, given its continuous prescription for treatment in typical tuberculosis patients, as employed in previous studies on drug toxicity [15,16]. Ethambutol initiation was defined as the first prescription fill date within the study period. Among the included patients, those with pre-existing ON or a diagnosis of multiple sclerosis were excluded to ensure that the analyzed cohort was devoid of pre-existing ON or other frequently encountered causes of optic neuritis/neuropathy. The cohort for final inclusion consisted of 204,598 ethambutol users and was followed from the date of ethambutol initiation until 31 December 2022 to evaluate the occurrence of ON. Figure 1 shows the inclusion/exclusion criteria and the number of subjects after applying the criteria. This research acquired approval from the Institutional Review Board of Hanyang University Hospital (file no. 2023-11-008) and adhered to the Declaration of Helsinki. The need for informed consent was waived because of the retrospective nature of our study and the utilization of anonymized data.

### 2.2. Exposure and Outcome Assessments

Information on age, sex, systemic diseases (such as diabetes, hypertension, hyperlipidemia, kidney disease, and liver disease), and medication history (particularly ethambutol and isoniazid use) were extracted from the HIRA database. Additionally, the potential causes of toxic ON, such as alcohol, tobacco, and nutritional deficiencies, were assessed. Given that there were no specific data on the uptake of alcohol or tobacco from the database, information on alcohol- or tobacco-related disorders or alcohol or tobacco dependence was identified on the basis of ICD-10 codes. Supplementary Table S1 shows a summary of the diagnostic codes used for this study. 

The occurrence of ON, which is the primary outcome of this study, was identified using the diagnostic codes of several categories of ON within the database, including codes for overall ON (ICD-10 codes H46, H47.7, and H47.2), optic neuritis/ON (ICD-10 code H46), and optic atrophy (ICD-10 code 47.2), and a concomitant prescription of ethambutol. Furthermore, visual impairment was assessed as a secondary outcome by using relevant diagnostic codes, including visual impairment or blindness (ICD-10 codes H54.0 and H54.4). 

### 2.3. Statistical Analysis

Descriptive statistics were used to summarize the demographic and clinical characteristics of the study population. Categorical variables were presented as frequencies and percentages, whereas continuous variables were reported as means with standard deviations or medians with interquartile ranges as appropriate. Multivariate logistic regression analyses were conducted to determine the adjusted odds ratios (ORs) for the risk of ON associated with each characteristic while controlling for potential confounding variables. Covariates for multivariate logistic regression analyses included age, sex, cumulative dose of ethambutol, diabetes, hypertension, hyperlipidemia, kidney disease, liver disease, malnutrition/nutritional deficiency, alcohol-related diseases, and tobacco-related diseases as covariates. These factors were selected on the basis of study results indicating their association to ON or their significance to the pharmacokinetics of ethambutol therapy (i.e., liver disease and kidney disease). The same analyses were performed to identify risk factors with visual impairment/blindness. Baseline (at the time of ethambutol initiation) predictive factors, which were associated with late ON development during the observation period, were also assessed, and hazard ratios (HRs) were calculated for each factor by adjusting for other confounding variables. The HRs of clinical variables such as age; sex; daily dose of ethambutol; indication of ethambutol use; and systemic diseases such as diabetes, hypertension, hyperlipidemia, kidney disease, liver disease, malnutrition/nutritional deficiency, alcohol-related diseases, and tobacco-related diseases at baseline were calculated using the Cox proportional hazards model. All analyses were performed using SAS version 9.4 (SAS Institute Inc., Cary, NC, USA), and a two-sided *p*-value of less than 0.05 was considered statistically significant.

## 3. Results

### 3.1. Demographic and Clinical Characteristics

Table 1 summarizes the demographic and clinical characteristics of the 204,598 ethambutol users included in this study. The cohort consisted of 118,427 males (57.9%) and 86,171 females (42.1%). The mean age was 60.1 years, and the majority of users were aged between 50 and 80 years, with 22.1%, 17.5%, and 17.2% of users in the 70–80, 50–59, and 60–69 age groups, respectively. A significant portion of the cohort had systemic diseases: 34.9% had diabetes mellitus, 45.4% had hypertension, 48.4% had hyperlipidemia, 18.8% had kidney disease, and 47.1% had liver disease. 

Regarding the indication for ethambutol use, the majority (76.7%) were treated for pulmonary tuberculosis. A significant proportion of users (88.4%) were on combined isoniazid therapy. The mean duration of ethambutol use was 12.1 months, and the mean daily dose of ethambutol was 902.9 mg.

### 3.2. Comparison of Clinical Characteristics between Patients with and without ON

Among the 204,598 ethambutol users, 5277 (2.6%) developed overall ON. Table 2 presents a comparison of the demographic and clinical characteristics between ethambutol users with ON and those without ON. Female patients were significantly more likely to develop ON (*p* < 0.001). The mean age of patients with ON was slightly higher at 60.8 years than that of patients without ON at 60.1 years (*p* = 0.010). Systemic diseases were more prevalent among patients with ON; these included diabetes mellitus, hypertension, hyperlipidemia, liver diseases, kidney diseases, and malnutrition or other nutritional deficiencies (all *p* < 0.001). Alcohol- and tobacco-related disorders were rare but showed a statistically significant difference between the two groups (*p* < 0.001 for both). In terms of ethambutol use, patients with ON had a higher cumulative dose than those without ON (*p* < 0.001). 

### 3.3. Risk Factors of Several ON Conditions and Visual Impairment

Table 3 presents the risk factors for various ON conditions in ethambutol users obtained by univariate and multivariate logistic regression analyses. For overall ON, female sex was a significant risk factor (OR = 1.51, 95% confidence interval [CI] = 1.43–1.60). Age also showed a slight but significant association (OR = 1.002, 95% CI = 1.000–1.003). Systemic diseases such as diabetes, hypertension, hyperlipidemia, kidney disease, liver disease, and malnutrition/nutritional disorders were all significantly associated with increased risk of overall ON. Interestingly, the concomitant use of isoniazid was associated with a decreased risk (OR = 0.78, 95% CI = 0.72–0.86).

For specific conditions such as optic neuritis/ON and optic atrophy, similar patterns were observed. Females had higher risk, and systemic conditions such as diabetes, hypertension, hyperlipidemia, kidney disease, liver disease, and malnutrition/nutritional disorders also posed significant risks. For optic atrophy, older age showed a strong association, and systemic diseases such as diabetes, hypertension, hyperlipidemia, kidney disease, liver disease, and malnutrition/nutritional disorders were significant risk factors. Remarkably, the protective effect of isoniazid was consistent across all conditions.

Table 4 presents the results of logistic regression analyses identifying risk factors for visual impairment among ethambutol users. In the multivariate analyses, age was significantly associated with an increased risk of visual impairment. Extrapulmonary tuberculosis and other indications for ethambutol use also showed significant associations. Among systemic diseases, diabetes, hypertension, hyperlipidemia, kidney disease, liver disease, and malnutrition or nutritional disorders were identified as significant risk factors. Whereas the concomitant use of isoniazid was significantly associated with a decreased risk of visual impairment (OR = 0.83, 95% CI = 0.71–0.97), it did not show a significant protective effect in the multivariate model. 

### 3.4. Baseline Predictive Factors of ON and Visual Impairment in Ethambutol Initiators

Table 5 shows the baseline predictive factors associated with ON and visual impairment among patients initiating ethambutol therapy, together with the HR from the univariate and multivariate analyses. For ON, female sex showed HRs of 1.467 and 1.478 in the univariate and multivariate analyses, respectively. The daily ethambutol dose was significantly associated with an increased risk of ON in the multivariate analysis. Factors such as diabetes and hyperlipidemia also showed significant associations with ON. For visual impairment, older age and systemic conditions such as diabetes and hypertension were identified as significant risk factors in the multivariate analyses.

## 4. Discussion

This nationwide study offers significant insights into the risk of ON and visual impairment during tuberculosis treatment and identified various risk factors, including demographic characteristics, systemic diseases, and other potential contributors to toxic ON. Specifically, female sex, older age, systemic diseases such as diabetes, hypertension, hyperlipidemia, kidney and liver diseases, and malnutrition or nutritional disorders significantly increased ON risk. Among them, older age and systemic diseases, including diabetes, hypertension, hyperlipidemia, kidney and liver diseases, and malnutrition, also increased the risk of visual impairment. Longer duration and higher cumulative doses of ethambutol were also linked to a higher incidence of ON, whereas concomitant isoniazid use reduced the risk. These findings suggest the complex mechanism of ON induced by ethambutol or the vulnerability of the optic nerve to diverse factors. This also emphasizes the clinical need for a thorough evaluation of risk factors for those receiving ethambutol therapy. Effective monitoring strategies are essential for mitigating the risk of vision-threatening adverse reactions in ethambutol users with risk factors.

Our findings reveal that ethambutol users were predominantly elderly, with a mean age exceeding 60 years. The prevalence of systemic diseases such as diabetes mellitus, hypertension, and hyperlipidemia underscores their common coexistence, which should be carefully evaluated in populations initiating ethambutol therapy for tuberculosis. The widespread use of combined isoniazid therapy in our cohort (88.4%) reflects the standard clinical practice of using combination therapy in tuberculosis management [17,18,19]. The diverse durations and doses of ethambutol highlight the heterogeneity of drug exposure and their potential effects on toxicity outcomes. These demographic and clinical characteristics provide crucial context for interpreting our cumulative incidence of ON (2.6%) and identified risk factors. These data also emphasize the need for tailored monitoring strategies and interventions to mitigate the risk of vision-threatening ON in high-risk populations.

There were significant differences between the clinical characteristics of ethambutol users who developed ON and those who did not. Female patients exhibited a higher susceptibility to ON, thus highlighting a gender disparity in risk, which was also found in other ocular toxicities due to systemic medications such as hydroxychloroquine retinopathy [20]. Moreover, patients who developed ON tended to be slightly older and have a greater prevalence of systemic diseases such as diabetes mellitus, hypertension, hyperlipidemia, liver disease, kidney disease, and malnutrition/nutritional deficiencies than their counterparts without ON. Notably, alcohol- and tobacco-related disorders also showed a notable difference between the groups. The findings also reveal that patients who developed ON had a higher cumulative dose and longer duration of ethambutol use, thus suggesting a potential dose-dependent relationship with ON development. Furthermore, a smaller proportion of patients with pulmonary tuberculosis were noted among those with ON, potentially suggesting differences in cumulative dose according to indications, as extrapulmonary tuberculosis is usually treated for a longer duration than pulmonary tuberculosis [18]. However, the significant differences observed from all explored characteristics in Table 2 can also be attributed to the large sample size and the heterogeneity of the two compared groups, as with a large number of subjects, it is easier to detect statistically significant differences. However, this also indicates that various confounding factors may have influenced the results. To address this, we performed multivariate analyses to account for these potential confounders (Table 3, Table 4 and Table 5).

Our risk factor analyses (Table 3), by adjusting several confounders, identified several risk factors. Most importantly, the association between higher cumulative doses of ethambutol further underscored the dose-dependent nature of ethambutol toxicity on optic nerve function. Furthermore, demographic features such as female and old age were associated with an increased risk of ON. This highlights the heightened need for monitoring and preventive strategies in female and old patients undergoing ethambutol therapy. Furthermore, advancing age was a significant risk factor for optic atrophy and visual impairment, thus highlighting the age-related vulnerability to more severe structural and visual outcomes among elderly patients, which was also confirmed in ethambutol users in a previous study [2]. Systemic diseases such as diabetes, hypertension, hyperlipidemia, liver disease, and kidney diseases were consistently identified as significant risk factors for ON and visual impairment. These findings emphasize the importance of considering comorbid conditions in risk assessment and management strategies of ON for ethambutol users. Interestingly, the protective effect of concomitant isoniazid use was consistently observed across different ON conditions but did not extend to reducing the risk of visual impairment.

In addition, malnutrition or nutritional disorders significantly increased the risk of ON in ethambutol users. Malnutrition or nutritional disorders significantly increase ON risk due to deficiencies in essential nutrients, namely, vitamins B1, B6, and B12, which are crucial for maintaining nerve health [21,22]. These neurotrophic vitamins play vital roles in neuronal function, and their deficiencies impair electron transport and reduce ATP production in nerve cells [22], exacerbating neurotoxic effects and increasing the susceptibility of the optic nerve to damage from toxic medications such as ethambutol.

Ethambutol has also been associated with inducing deficiencies in these neurotrophic vitamins, further increasing the risk of ON in malnourished individuals. Accordingly, some experts recommend a combination of vitamins, specifically Vitamin B12, along with B1 and B6, for the primary prevention of optic neuropathy in ethambutol users [23]. However, isoniazid, primarily linked to peripheral neuropathy rather than ON, is routinely administered with pyridoxine (vitamin B6) to prevent neurotoxic side effects, as some guidelines suggest [6,23]. This supplemental pyridoxine may also help mitigate the risk of EON by maintaining adequate vitamin B6 levels, thereby offering some protection to the optic nerve. Therefore, the confounding effect of vitamin supplements should be carefully considered and adjusted; however, assessing patient uptake of vitamin B6 from our health claims database is challenging, as it may come from over-the-counter supplements. Additionally, the presence of numerous codes for pyridoxine in our health claims databases (>1000), along with limitations on data retrieval from the HIRA database, complicated our comprehensive analysis of the effect of vitamin B6 on determining the true relationship between isoniazid and ethambutol-induced optic nerve toxicity. This should be carefully addressed in future studies in terms of mechanisms of optic nerve damage caused by ethambutol and its interaction with isoniazid and nutritional deficiency.

Although ethambutol is well documented for its potential to cause ON, the effect of isoniazid is often underrecognized. Isoniazid-induced ON, although less common than ethambutol-induced cases, can present similar symptoms such as decreased visual acuity, color vision deficits, and central scotomas [6,24]. The concomitant use of isoniazid and ethambutol has been believed to have an additive toxic effect, thus exacerbating the risk of ON [6]. However, our findings indicate that there was no synergistic effect between the two, suggesting that ethambutol and isoniazid may involve different mechanisms in causing ON. Mechanistically, EON is attributed to its metal chelation effects, particularly on copper and zinc, which disrupt mitochondrial function and cause cellular energy depletion. Zinc chelation also inhibits lysosomal activation, thus affecting cellular waste processing [1,25]. Animal studies indicate that ethambutol-induced zinc deficiency may lead to myelin destruction and glial cell proliferation in optic nerves [26,27]. By contrast, the mechanism of isoniazid-associated ON is likely different from that of ethambutol because isoniazid is not known for its metal chelating properties. 

Notably, by comparing the daily dose and duration of ethambutol use between those treated with ethambutol without isoniazid and those with ethambutol and isoniazid, we found that the combined isoniazid therapy significantly reduced ethambutol use duration. There were significant differences in the mean duration of ethambutol therapy (18.0 ± 18.6 months in those without isoniazid and 11.3 ± 10.2 months in those with isoniazid; *p* < 0.001). Although there were slight differences in the mean daily dose between the two groups (868.3 ± 202.7 mg in those without isoniazid vs. 907.5 ± 191.7 mg in those with combined isoniazid), the shorter duration of ethambutol in those treated with combined isoniazid and ethambutol therapy might explain the reduced risk of EON. However, to adjust for the effect of combined isoniazid on ethambutol dose reduction, we included the cumulative dose of ethambutol as a covariate in the multivariate analyses. Consequently, the protective effect of combined isoniazid on visual impairment was insignificant in the multivariate analyses (Table 4) but still significant for ON (Table 3). Accordingly, the protective effect of isoniazid should be validated in future studies, with careful consideration and adjustment of its effects on ethambutol dose reduction.

The findings on several key baseline factors associated with the development of ON and visual impairment have additional practical implications in treatment and monitoring decisions for patients initiating ethambutol therapy for tuberculosis (Table 5). Female sex and older age are consistent predictors of an increased risk of ON, as evidenced by significant HRs in both the univariate and multivariate analyses. The association with daily ethambutol dose further highlights the importance of monitoring toxicity in a dose-dependent manner through pharmacovigilance. Systemic conditions such as diabetes and hyperlipidemia also emerged as significant predictive factors, indicating the importance of considering these comorbidities when planning baseline monitoring. 

However, our study has a few limitations. First, the retrospective design and reliance on health claims databases of this study inherently introduce typical biases such as selection bias and misclassification errors. For example, the use of diagnostic codes for identifying ON cases could lead to misclassification or underreporting, thus potentially affecting the accuracy of our incidence estimates, particularly given the lack of specificity in certain ON categories that require further validation. The daily dose divided by body weight should be considered to define the risk of retinopathy as a measure of drug exposure, as in previous studies [1,4]; however, information on body weight was not available in the HIRA database. Furthermore, due to the absence of pharmacogenetic data from the HIRA database, our study could not account for genetic factors contributing to ON development. As genetic predispositions, such as mitochondrial mutations associated with LHON or hereditary optic neuropathy, may affect the risk of EON [28,29,30], this is another limitation of our study. Future research incorporating pharmacogenetic analyses may provide more comprehensive risk factor analyses, thereby enhancing the precision of risk assessments and personalized interventions. Additionally, considering that the study population consisted exclusively of Koreans, our findings may not be fully generalizable to other populations with different healthcare systems and tuberculosis epidemiological profiles. The inclusion of several ON categories might lack specificity, for which further validation is required to confirm our findings. Furthermore, the absence of quantitative data on alcohol or tobacco use from our database limits our ability to definitively assess their association with ethambutol toxicity. Finally, we acknowledge that various conditions affecting the pharmacokinetics or distribution of ethambutol in the CNS and neurological diseases such as meningitis could be potential risk factors for EON, which we did not evaluate. These limitations underscore the need for a cautious interpretation of our findings and suggest avenues for improving future research in this area.

## 5. Conclusions

In conclusion, this nationwide study significantly enhances our understanding of the risk factors associated with ON and visual impairment in ethambutol users. Our findings show the significant association of demographic characteristics, systemic diseases, and drug exposure with ON and visual impairment in ethambutol users. This study also highlights the potential protective effects of isoniazid, although further investigation is needed to elucidate the underlying mechanisms and interactions between these drugs. This information needs to be utilized to refine risk assessment and monitoring schemes for ON and to enhance the ocular safety of ethambutol treatment in patients with tuberculosis.

## Figures and Tables

**Table 1:** Demographic and clinical information of ethambutol users included in this study.

Characteristics	Overall Users (*n* = 204,598)
Sex	
Male/female	118,427 (57.9%):86,171 (42.1%)
Mean age (±SD), years	60.1 ± 19.0
<30	17,560 (8.6%)
30–39	16,453 (8.0%)
40–49	23,454 (11.5%)
50–59	35,707 (17.5%)
60–69	35,168 (17.2%)
70–80	45,152 (22.1%)
≥80	31,104 (15.2%)
Systemic diseases	
Diabetes mellitus	71,392 (34.9%)
Hypertension	92,843 (45.4%)
Hyperlipidemia	99,016 (48.4%)
Kidney disease	38,405 (18.8%)
Liver disease	96,261 (47.1%)
Malnutrition or other nutritional deficiency	51,150 (25.0%)
Alcohol-related disorders	121 (0.1%)
Tobacco-related disorders	194 (0.1%)
Indication for ethambutol use	
Pulmonary tuberculosis	156,962 (76.7%)
Extrapulmonary tuberculosis	22,722 (11.1%)
Others	24,914 (12.2%)
Combined use of isoniazid (%)	180,874 (88.4%)
Mean duration of ethambutol use (±SD), months	12.1 ± 11.7
Less than 3 months	42,636 (20.8%)
3–6 months	27,315 (13.4%)
6 months–1 year	44,022 (21.5%)
1–1.5 years	46,603 (22.8%)
1.5–2 years	22,212 (10.9%)
2 years or longer	21,810 (10.7%)
Mean daily dose of ethambutol (±SD), mg/day	902.9 ± 193.5
Less than 750 mg	11,266 (5.5%)
750–1000 mg	137,517 (67.2%)
1000–1250 mg	51,877 (25.4%)
1250 mg or greater	3938 (1.9%)
Mean cumulative dose of ethambutol (±SD), g	315.7 ± 301.1
Less than 200 g	83,270 (40.7%)
200–400 g	60,021 (29.3%)
400–600 g	36,239 (17.7%)
600 g or greater	25,068 (12.3%)

SD, standard deviation.

**Table 2:** Demographic and clinical characteristics of patients with and without overall optic neuropathy.

Characteristics	Without Optic Neuropathy (*n* = 199,321)	With Optic Neuropathy (*n* = 5277)	*p*-Value
Sex			
Female (%)	83,455 (41.9%)	2716 (51.5%)	<0.001
Mean age (±SD), years	60.1 ± 19.1	60.8 ± 17.5	0.010
Systemic diseases			
Diabetes mellitus (%)	69,102 (34.7%)	2290 (43.4%)	<0.001
Hypertension (%)	90,034 (45.2%)	2809 (53.2%)	<0.001
Hyperlipidemia (%)	95,686 (48.0%)	3330 (63.1%)	<0.001
Liver diseases (%)	37,104 (18.6%)	1301 (24.7%)	<0.001
Kidney diseases (%)	93,291 (46.8%)	2970 (56.3%)	<0.001
Malnutrition or other nutritional deficiency	49,381 (24.8%)	1769 (33.5%)	<0.001
Alcohol-related disorders (F10, Z72.1, T51)	118 (0.1%)	3 (0.1%)	<0.001
Tobacco-related disorders (F17, Z72.0, T65.2	188 (0.1%)	6 0.1(%)	<0.001
Indication for ethambutol use			
Pulmonary tuberculosis (%)	153,228 (76.9%)	3734 (70.8%)	<0.001
Cumulative dose of ethambutol (± SD), g	312.9 ± 297.8	418.2 ± 392.4	<0.001
Mean (median) duration of ethambutol use, months	12.0 (10.8)	15.9 (12.3)	<0.001
Mean daily dose of ethambutol (± SD), mg/day	902.5 ± 193.5	919.3 ± 191.7	<0.001

**Table 3:** Risk factors for several optic neuropathy conditions on logistic regression analyses in ethambutol users.

Factors	Overall Optic Neuropathy				Optic Neuropathy/Optic Neuritis				Optic Atrophy			
	Univariate		Multivariate		Univariate		Multivariate		Univariate		Multivariate	
	OR (95% CI)	*p*	OR (95% CI)	*p*	OR (95% CI)	*p*	OR (95% CI)	*p*	OR (95% CI)	*p*	OR (95% CI)	*p*
Sex *	1.47 (1.39–1.56)	<0.001	1.51 (1.43–1.60)	<0.001	1.58 (1.49–1.69)	<0.001	1.50 (1.42–1.59)	<0.001	1.33 (1.18–1.50)	<0.001	1.50 (1.42–1.58)	<0.001
Age	1.002 (1.000–1.003)	0.017	0.998 (0.996–1.000)	0.018	0.999 (0.997–1.001)	0.206			1.02 (1.01–1.02)	<0.001	0.997 (0.995–0.999)	0.002
Monitoring period, months	1.02 (1.02–1.02)	<0.001	1.00 (1.00–1.01)	0.335	1.02 (1.02–1.02)	<0.001	1.00 (1.00–1.00)	0.391	1.01 (1.01–1.01)	<0.001	0.99 (0.98–0.99)	<0.001
Indications for ethambutol use ^†^		<0.001		0.002		<0.001		0.001		0.240		
Extrapulmonary Tb	1.30 (1.19–1.41)		1.14 (1.05–1.24)		1.44 (1.31–1.58)		1.15 (1.06–1.25)		0.90 (0.73–1.10)			
Others	1.44 (1.34–1.56)		1.11 (1.02–1.22)		1.62 (1.48–1.76)		1.11 (1.01–1.21)		1.11 (0.93–1.33)			
Cumulative dose	1.001 (1.001–1.001)	<0.001	1.001 (1.000–1.001)	0.003	1.001 (1.001–1.001)	<0.001	1.001 (1.000–1.001)	0.002	1.000 (1.000–1.001)	<0.001	1.001 (1.001–1.002)	<0.001
Concomitant use of isoniazid	0.65 (0.61–0.70)	<0.001	0.78 (0.72–0.86)	<0.001	0.57 (0.52–0.62)	<0.001	0.79 (0.72–0.86)	<0.001	0.98 (0.81–1.18)	<0.001	0.75 (0.70–0.81)	<0.001
Diabetes	1.45 (1.37–1.53)	<0.001	1.14 (1.07–1.22)	<0.001	1.30 (1.22–1.38)	<0.001	1.13 (1.06–1.21)	<0.001	2.28 (2.02–2.57)	<0.001	1.14 (1.07–1.22)	<0.001
Hypertension	1.38 (1.31–1.46)	<0.001	1.12 (1.05–1.20)	0.001	1.25 (1.17–1.33)	<0.001	1.08 (1.02–1.15)	0.011	2.26 (1.99–2.56)	<0.001	1.12 (1.05–1.20)	0.001
Hyperlipidemia	1.85 (1.75–1.96)	<0.001	1.47 (1.38–1.58)	<0.001	1.74 (1.63–1.86)	<0.001	1.47 (1.38–1.57)	<0.001	2.47 (2.16–2.81)	<0.001	1.48 (1.39–1.58)	<0.001
Kidney disease	1.43 (1.34–1.53)	<0.001	1.19 (1.12–1.28)	<0.001	1.36 (1.26–1.46)	<0.001	1.19 (1.11–1.27)	<0.001	1.90 (1.66–2.16)	<0.001	1.20 (1.12–1.29)	<0.001
Liver disease	1.46 (1.39–1.55)	<0.001	1.47 (1.38–1.58)	<0.001	1.38 (1.29–1.47)	<0.001	1.15 (1.09–1.22)	<0.001	1.84 (1.62–2.08)	<0.001	1.14 (1.08–1.21)	<0.001
Malnutrition/nutritional disorders	1.53 (1.45–1.62)	<0.001	1.27 (1.19–1.34)	<0.001	1.56 (1.45–1.66)	<0.001	1.26 (1.19–1.34)	<0.001	1.58 (1.40–1.80)	<0.001	1.27 (1.20–1.35)	<0.001
Alcohol-related disorders	0.96 (0.31–3.02)	0.946			0.87 (0.22–3.51)	0.847			1.60 (0.23–11.39)	0.638		
Tobacco-related disorders	1.21 (0.54–2.72)	0.652			1.64 (0.73–3.70)	0.233			N/A	N/A		

N/A = not applicable. * Male as reference. **^†^** Pulmonary tuberculosis as reference.

**Table 4:** Risk factors for visual impairment on logistic regression analyses in ethambutol users.

Factors	Univariate		Multivariate	
	OR (95% CI)	*p*	OR (95% CI)	*p*
Sex *	1.05 (0.94–1.17)	0.417		
Age	1.02 (1.01–1.02)	<0.001	1.01 (1.01–1.02)	<0.001
Monitoring period, months	1.01 (1.01–1.01)	<0.001	1.00 (0.98–1.02)	0.858
Indications for ethambutol use ^†^		<0.001		<0.001
Extrapulmonary Tb	1.26 (1.07–1.48)		1.32 (1.12–1.56)	
Others	1.42 (1.22–1.65)		1.34 (1.13–1.58)	
Cumulative dose	1.000 (1.000–1.001)	<0.001	1.000 (1.000–1.001)	0.214
Concomitant use of isoniazid	0.83 (0.71–0.97)	0.018	0.97 (0.81–1.16)	0.698
Diabetes	2.19 (1.97–2.44)	<0.001	1.33 (1.17–1.50)	<0.001
Hypertension	2.39 (2.13–2.67)	<0.001	1.40 (1.22–1.60)	<0.001
Hyperlipidemia	2.47 (2.20–2.77)	<0.001	1.53 (1.34–1.75)	<0.001
Kidney disease	2.03 (1.81–2.28)	<0.001	1.37 (1.21–1.55)	<0.001
Liver disease	1.68 (1.51–1.88)	<0.001	1.18 (1.05–1.33)	0.006
Malnutrition or nutritional disorders	1.71 (1.53–1.92)	<0.001	1.30 (1.16–1.46)	<0.001
Alcohol-related disorders	3.86 (1.23–12.17)	0.021	3.02 (0.95–9.55)	0.061
Tobacco-related disorders	0.79 (0.11–5.61)	0.810		

* Male as reference. ^†^ Pulmonary tuberculosis as reference.

**Table 5:** Baseline (at the time of ethambutol initiation) predictive factors of optic neuropathy and visual impairment and their hazard ratios (HRs).

Factors	Optic Neuropathy				Visual Impairment			
	Univariate		Multivariate		Univariate		Multivariate	
	HR (95% CI)	*p*	HR (95% CI)	*p*	HR (95% CI)	*p*	HR (95% CI)	*p*
Sex *	1.467 (1.390–1.548)	<0.001	1.478 (1.399–1.563)	<0.001	0.692 (0.548–0.873)	0.002	0.630 (0.498–0.797)	<0.001
Age	1.002 (1.001–1.004)	0.002	1.000 (0.998–1.002)	0.950	1.031 (1.024–1.038)	<0.001	1.022 (1.014–1.030)	<0.001
Daily dose	1.000 (1.000–1.001)	<0.001	1.001 (1.000–1.001)	<0.001	1.000 (0.999–1.000)	0.150		
Indications for ethambutol use ^†^		<0.001		<0.001		0.143		
Extrapulmonary Tb	1.296 (1.195–1.405)		1.204 (1.109–1.307)		0.996 (0.695–1.427)			
Others	1.458 (1.354–1.571)		1.151 (1.059–1.251)		1.358 (0.998–1.846)			
Concomitant use of isoniazid	0.630 (0.586–0.675)	<0.001	0.669 (0.620–0.723)	<0.001	1.018 (0.726–1.430)	0.916		
Diabetes	1.177 (1.115–1.243)	<0.001	1.147 (1.075–1.223)	<0.001	2.088 (1.669–2.613)	<0.001	1.311 (1.016–1.692)	0.037
Hypertension	1.081 (1.024–1.140)	0.005	0.952 (0.888–1.020)	0.160	2.948 (2.312–3.759)	<0.001	1.841 (1.372–2.471)	<0.001
Hyperlipidemia	1.302 (1.231–1.377)	<0.001	1.245 (1.163–1.333)	<0.001	1.591 (1.263–2.006)	<0.001	0.847 (0.645–1.113)	0.233
Kidney disease	1.108 (1.042–1.177)	0.001	1.003 (0.940–1.071)	0.918	1.533 (1.213–1.938)	<0.001	1.047 (0.816–1.342)	0.720
Liver disease	1.074 (1.017–1.134)	0.011	0.950 (0.893–1.010)	0.103	1.488 (1.183–1.873)	0.001	1.056 (0.819–1.361)	0.674
Malnutrition or nutritional disorders	1.072 (1.006–1.142)	0.032	0.985 (0.922–1.051)	0.641	1.540 (1.213–1.956)	<0.001	1.200 (0.938–1.534)	0.147

* Male as reference. **^†^** Pulmonary tuberculosis as reference.

## Data Availability

Data and materials can be requested by e-mail and will be provided after consultation with the IRB.

## Supplementary Materials

The following supporting information can be downloaded at: https://www.mdpi.com/article/10.3390/toxics12080549/s1, Table S1: Definitions and corresponding ICD-10 and Korean Classification of Disease (KCD)-7 or -8 codes used in this study.
