# Supplementary material for: Risk Factors of Optic Neuropathy in Ethambutol Users: Interaction with Isoniazid and Other Associated Conditions of Toxic Optic Neuropathy
Source: Toxics. 2024 Jul 30;12(8):549. doi: 10.3390/toxics12080549 (PMC11359443; doi:10.3390/toxics12080549)
Supplement: Supplementary file 1 [file toxics-12-00549-s001.zip › toxics-3117752-supplementary.pdf]

Table S1. Definitions and corresponding ICD-10 and Korean Classification of Disease (KCD)-7 or -8 codes used in this study

Category	ICD-10 codes	KCD codes	Specific conditions
	H46	H46	Optic neuritis/neuropathy (except ischemic cause)
Overall optic neuropathy	H47.09 and H47.9	H47.7	Unspecified disorder of visual pathways
	H47.2	H47.2	Optic atrophy
Optic neuritis/neuropathy	H46	H46	Optic neuritis/neuropathy (except ischemic cause)
Optic atrophy	H47.2	H47.2	Optic atrophy
Visual impairment	H54	H54	Visual impairment including blindness
